# Complete genome analysis of a virulent *Vibrio scophthalmi* strain VSc190401 isolated from diseased marine fish half-smooth tongue sole, *Cynoglossus semilaevis*

**DOI:** 10.1186/s12866-020-02028-7

**Published:** 2020-11-11

**Authors:** Zheng Zhang, Yong-xiang Yu, Yin-geng Wang, Xiao Liu, Li-fang Wang, Hao Zhang, Mei-jie Liao, Bin Li

**Affiliations:** 1 Key Laboratory of Maricultural Organism Disease Control, Ministry of Agriculture and Rural Affairs, Yellow Sea Fisheries Research Institute, Chinese Academic of Fishery Sciences, Qingdao, Shandong 266071 PR China; 2 Laboratory for Marine Fisheries Science and Food Production Processes, Pilot National Laboratory for Marine Science and Technology (Qingdao), Qingdao, Shandong 266237 PR China

**Keywords:** *Vibrio scophthalmi*, Bacterial genome, Pathogenicity, Virulence factors, Drug resistance

## Abstract

**Background:**

*Vibrio scophthalmi* is an opportunistic bacterial pathogen, which is widely distributed in the marine environment. Earlier studies have suggested that it is a normal microorganism in the turbot gut. However, recent studies have confirmed that this bacterial strain can cause diseases in many different marine animals. Therefore, it is necessary to investigate its whole genome for better understanding its physiological and pathogenic mechanisms.

**Results:**

In the present study, we obtained a pathogenic strain of *V. scophthalmi* from diseased half-smooth tongue sole (*Cynoglossus semilaevis*) and sequenced its whole genome. Its genome contained two circular chromosomes and two plasmids with a total size of 3,541,838 bp, which harbored 3185 coding genes. Among these genes, 2648, 2298, and 1915 genes could be found through annotation information in COG, Blast2GO, and KEGG databases, respectively. Moreover, 10 genomic islands were predicted to exist in the chromosome I through IslandViewer online system. Comparison analysis in VFDB and PHI databases showed that this strain had 334 potential virulence-related genes and 518 pathogen-host interaction-related genes. Although it contained genes related to four secretion systems of T1SS, T2SS, T4SS, and T6SS, there was only one complete T2SS secretion system. Based on CARD database blast results, 180 drug resistance genes belonging to 27 antibiotic resistance categories were found in the whole genome of such strain. However, there were many differences between the phenotype and genotype of drug resistance.

**Conclusions:**

Based on the whole genome analysis, the pathogenic *V. scophthalmi* strain contained many types of genes related to pathogenicity and drug resistance. Moreover, it showed inconsistency between phenotype and genotype on drug resistance. These results suggested that the physiological mechanism seemed to be complex.

**Supplementary Information:**

The online version contains supplementary material available at 10.1186/s12866-020-02028-7.

## Background

*Vibrio scophthalmi* was first isolated from larval turbot (*Scophthalmus maximus*) intestine by Spanish scientists in 1997 and identified as a new species of *Vibrio* genus [1]. In terms of phylogenetic status, this bacterium shares very high gene sequence similarity and amino acid identity with *Vibrio ichthyoenteri* [2]. Moreover, they also have similar physiological and biochemical characteristics [3]. *V. ichthyoenteri* is considered as a causative agent of Japanese flounder intestinal disease (*Paralichthys olivaceus*). Earlier studies have suggested that *V. scophthalmi* is a type of common organism colonized in the turbot (*Scophthalmus maximus*) gut, which is not pathogenic and has certain host specificity for turbot [4]. Subsequently, this bacterium is successively isolated from diseased *Paralichthys olivaceus* [5], *Paralichthys dentatus* [6], *Dentex dentex* [7], *Ruditapes philippinarum* [8] and *Thunnus maccoyii* [9], supporting its pathogenicity to aquatic animals.

Current studies have proved that *V. scophthalmi* is an opportunistic pathogen [5], and it generally does not cause diseases when it is in the intestine of healthy fish. However, when the fish are subjected to environmental stress and their immunity is weakened, they are easily infected by this bacterial strain, leading to disease or death. For example, if the water temperature rises to 20 °C, eel (*Anguilla japonica*) is easy to be infected by *V. scophthalmi*, which results in symptoms of severe enteritis and ascites, leading to high mortality [10]. *V. scophthalmi* can be a secondary agent to significantly increase the mortality of diseased fish after the infections of other pathogens [5]. Scientists have confirmed that *V. scophthalmi* is one of the main pathogenic bacteria for Japanese flounder (*P. olivaceus*) cultured in Jeju area of Korean [11]. It is also a major pathogen of cultured turbot in China [12]. The typical symptoms of *V. scophthalmi-*infected fish include body surface blackening, ascites, enteritis, and internal organ hyperaemia [5, 13], leading to 30 to 90% mortality of infected fish and huge economic losses.

It is generally believed that extracellular substances, including proteases and exotoxins, are the key virulence factors for most pathogenic Vibrio bacteria [14]. However, the pathogenic mechanism of *V. scophthalmi* still remains largely unexplored until now. Previous studies have confirmed that the extracellular products of *V. scophthalmi* show a variety of protease activities, such as naphthol-AS-BI-phosphohydrolase, lipase, gelatinase, and leucine arylamidase [15], while no hemolytic activity or cytotoxic effect has been reported. Besides, some studies have shown that *V. scophthalmi* exhibits resistance to a variety of antibiotics [16, 17].

In the present study, a strain of *V. scophthalmi* was isolated from diseased half-smooth tongue sole (*Cynoglossus semilaevis*), and its pathogenicity to fish was verified by artificial infection experiment. Subsequently, the whole genome of this strain was sequenced and analyzed in detail. Collectively, our findings provided valuable insights into molecular mechanisms underlying the pathogenicity of *V. scophthalmi* and drug resistance.

## Results and discussion

### The pathogenicity of strain VSc190401

**Fig. 1:**
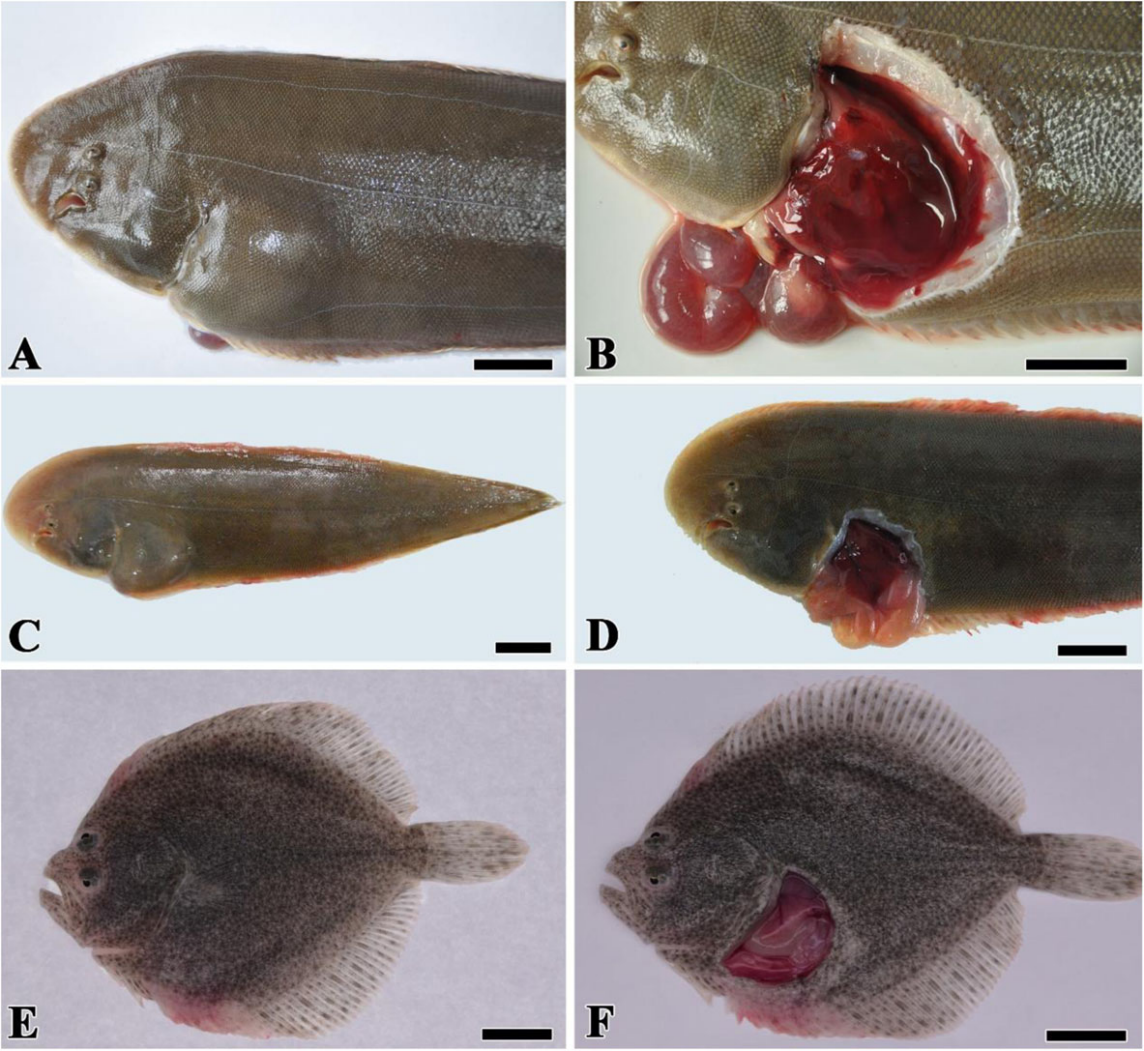
The symptoms of diseased fish with natural infection and artificial infection *V. scophthalmi* strain VSc190401. **a**: Naturally infected half-smooth tongue sole had obvious abdominal lump. **b**: Naturally infected half-smooth tongue sole showed serious internal organ hyperemia and enteritis. **c**: Artificially infected half-smooth tongue soles showed the same symptoms of abdominal limp. **d**: Artificially infected half-smooth tongue soles appeared the same internal organ lesions as the naturally infected case. **e**: Artificially infected turbot did not appear abdominal lump. **f**: Artificially infected turbot showed serious organ hyperemia and enteritis. Bar = 2 cm

The diseased half-smooth tongue sole naturally infected by strain VSc190401 showed an apparent abdominal lump (Fig. 1a). After dissection, effusion flowed out from the abdominal cavity. The internal organs exhibited serious hyperemia, and the intestinal tract became thin and transparent, which was filled with a large amount of effusion and white pus (Fig. 1b). The mortality rate of this case was more than 40%.

During the artificial challenge test, the fish in the negative control group and blank control group remained healthy and showed no symptoms. The half-smooth tongue sole artificially infected in the experiment group with strain VSc190401 also showed the same symptoms as the naturally infected fish, such as effusion in the abdominal cavity, internal organ hyperemia, and thin and transparent intestine. The artificially infected turbot did not appear abdominal lump, while the organ hyperemia and intestinal inflammation were observed. Artificial infection confirmed that the *V. scophthalmi* strain VSc190401 had strong pathogenicity to fish.

### Genomic information of strain VSc190401

**Fig. 2:**
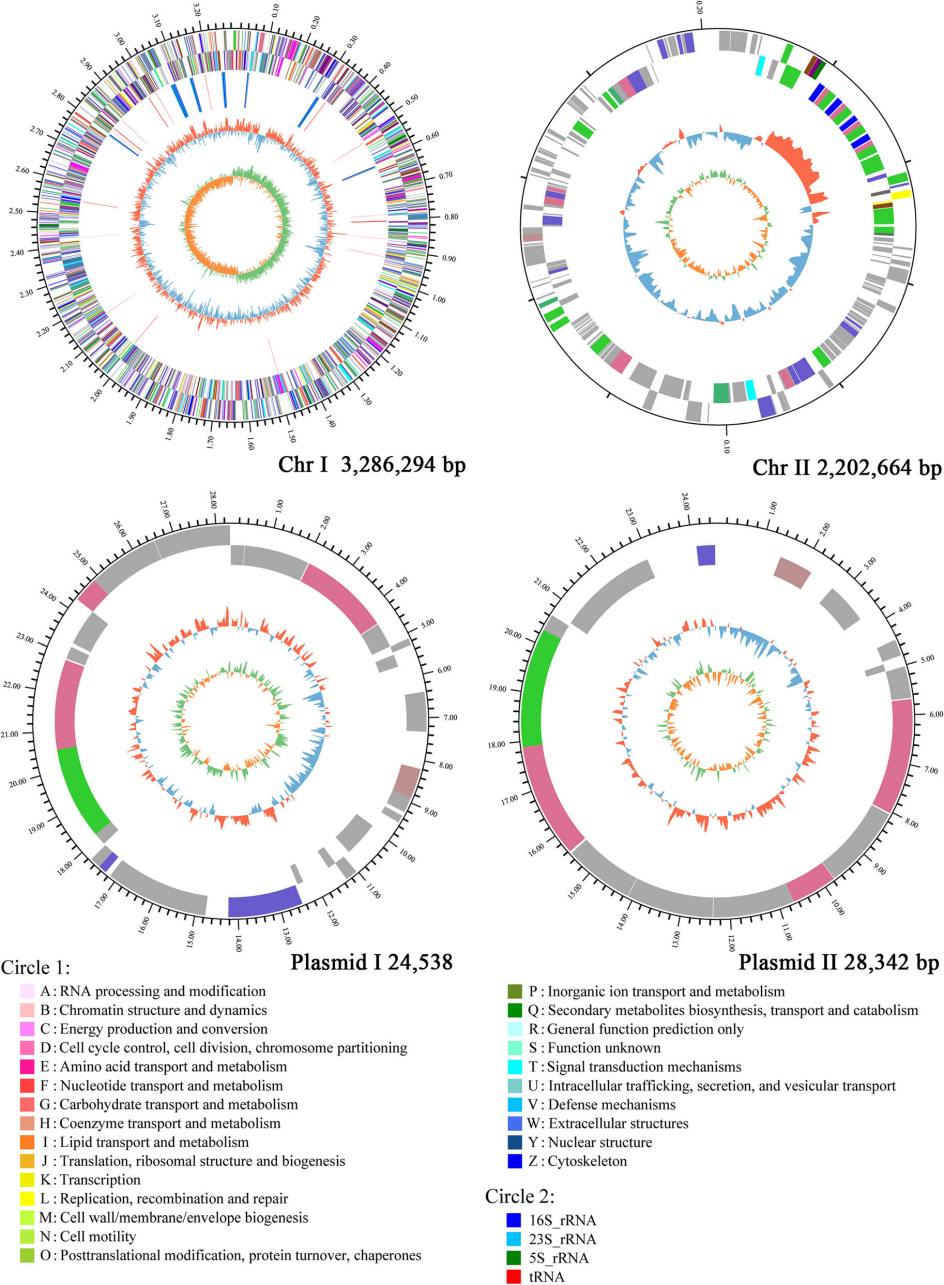
Circular genome maps of *V. scophthalmi* strain VSc190401. Note: The outermost circle is the identification of genome size. The second and the third circle are the CDSs on the positive and negative strands, respectively, and different colors indicate the different functional annotations of CDSs in the COG database. The fourth circle is rRNA and tRNA. The fifth circle is the GC content, the red part outside indicates that the GC content of the region is higher than the average GC content of the whole genome, the blue part inward indicates that the GC content of the region is lower than the average GC content of the whole genome, and the higher peak value means the greater difference from the average GC content. The innermost circle is the GC-Skew value, and its algorithm is $\frac{\mathrm{G}-\mathrm{C}}{\mathrm{G}+\mathrm{C}}$, which can assist to determine the leading strand and lagging strand. In general, the leading strand GC-skew > 0 and the lagging strand GC-skew < 0. The green part outside means GC-skew > 0, the orange part inward means GC-skew < 0, and the higher peak value means larger value. The legend circle1 is the functional classification in the COG database, and the legend circle2 is a different RNA classification

After assembly, the size of the whole genome of *V. scophthalmi* strain VSc190401 was 3,541,838 bp, including two circular chromosomes (Chr I 3,286,294 bp and Chr II 202,664 bp) with an approximate GC content of 45.00 and 45.37%, respectively, and two plasmids (plasmid I 24,538 and plasmid II 28,342 bp) with an approximate GC content of 43.32 and 43.41%, respectively. The strain contained 3185 coding genes, among which Chr I consisted of 2943 CDSs, 104 tRNA genes, and 37 rRNA genes. Chr II contained 188 CDSs. Plasmid I contained 32 CDSs, and plasmid II contained 22 CDSs. Figure 2 shows the genome information.

### Phylogenetic analyses

**Fig. 3:**
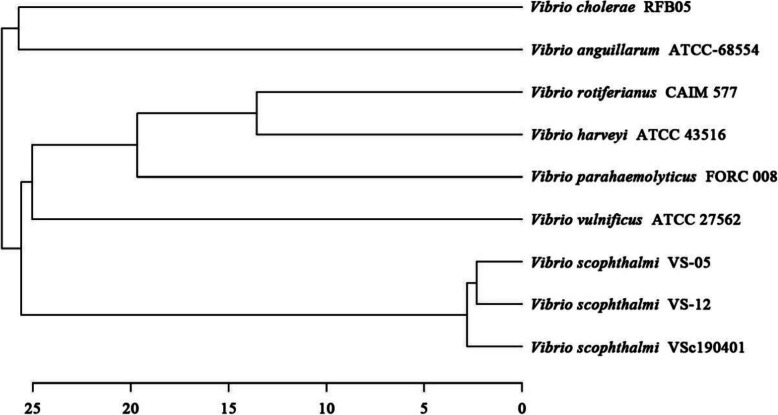
Phylogenetic tree analysis based on ANI values of *V. scophthalmi* strain VSc190401 and the complete genomes of eight bacterial strains downloaded from the NCBI database. The scale represents genetic distance

ANI analysis showed that *V. scophthalmi* strain VSc190401 was clustered with *V. scophthalmi* VS-12 and VS-05 in the whole genome of eight selected strains, and there were some genetic differences (Fig. 3). The annotation information in the NCBI database indicated that *V. scophthalmi* VS-12 and VS-05 strains were all isolated from Japanese flounder cultured in Korea, containing three and two plasmids in addition to two chromatins, respectively. Based on the ANI analysis, the whole genome of *V. scophthalmi* strain VSc190401 was found to be genetically different compared with Korean isolate strains VS-12 and VS-05, suggesting that there were some differences in host diversity or pathogenicity between Chinese and Korean isolates.

### Functional annotation

**Fig. 4:**
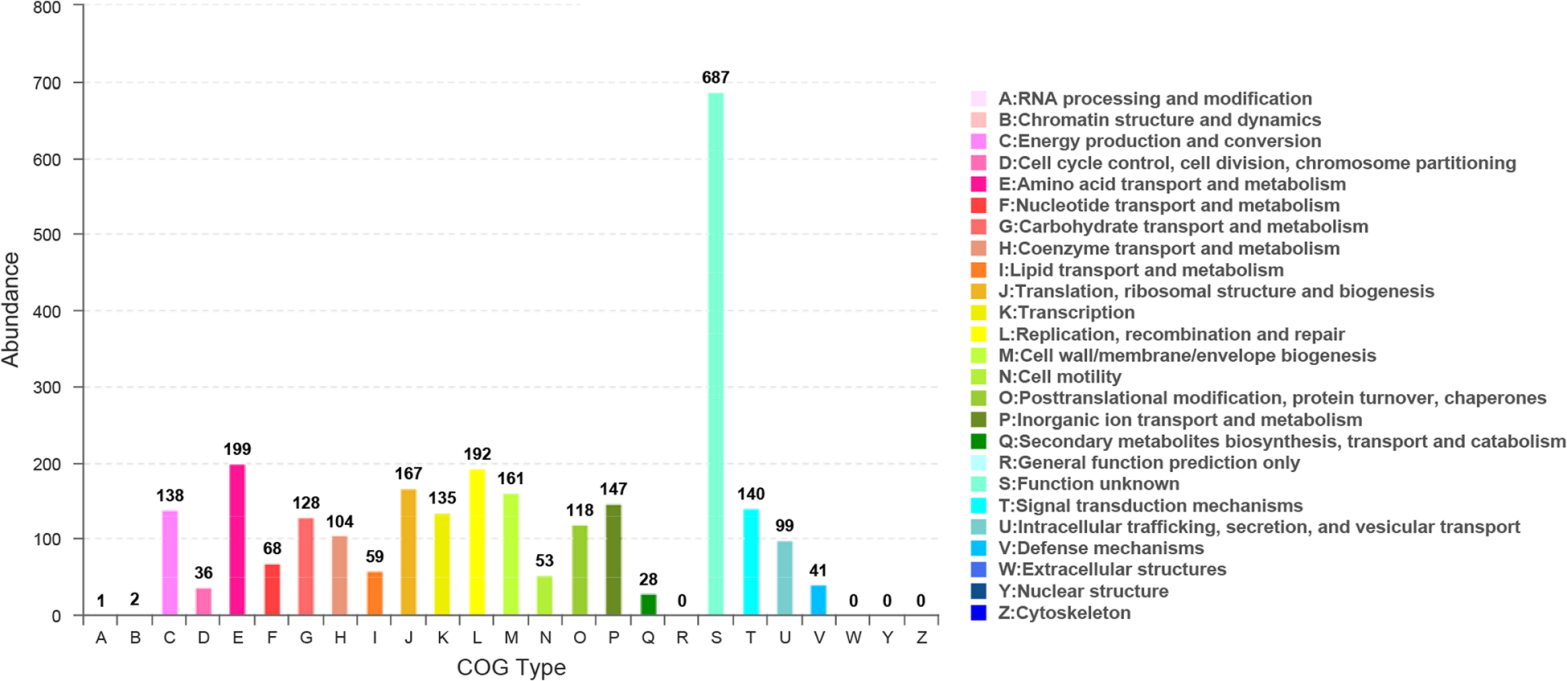
COG functional annotation of CODs in the whole genome of *V. scophthalmi* strain VSc190401

COG annotation results showed that 2648 genes were annotated into 22 types of genes, accounting for 83.14% of total genes in *V. scophthalmi* strain VSc190401. The number of each type of gene was as follows: one A-type gene (RNA processing and modification), two B-type genes (chromatin structure and dynamics), 138 C-type genes (energy production and conversion), 36 D-type genes (cell cycle control, cell division, chromosome partitioning), 199 E-type genes (amino acid transport and metabolism), 68 F-type genes (nucleotide transport and metabolism), 128 G-type genes (carbohydrate transport and metabolism), 104 H-type genes (coenzyme transport and metabolism), 59 I-type genes (lipid transport and metabolism), 167 J-type genes (translation, ribosomal structure and biogenesis), 135 K-type genes (transcription), 192 L-type genes (replication, recombination and repair), 161 M-type genes (cell wall/membrane/envelope biogenesis), 53 N-type genes (cell motility), 118 O-type genes (posttranslational modification, protein turnover, chaperones), 147 P-type genes (inorganic ion transport and metabolism), 28 Q-type genes (secondary metabolites biosynthesis, transport and catabolism), 687 S-type genes (function unknown), 140 T-type genes (signal transduction mechanisms), 99 U-type genes (intracellular trafficking, secretion, and vesicular transport), and 41 V-type genes (defense mechanisms) (Fig. 4). Supplementary Table S1 lists all COG functional annotation information.

**Fig. 5:**
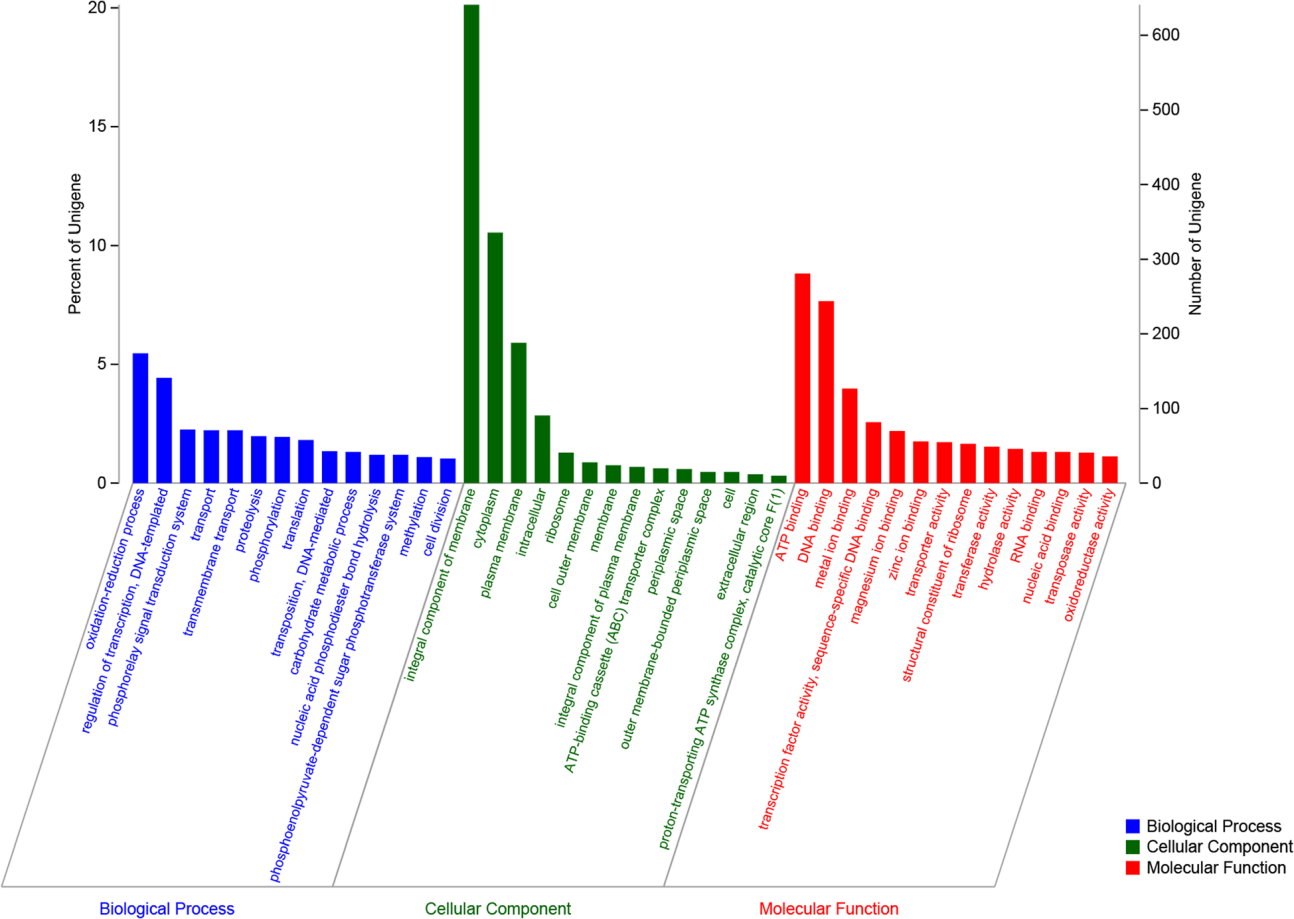
GO functional annotation of CODs in the whole genome of *V. scophthalmi* strain VSc190401

The functional annotation results in the GO database showed that 2298 genes were annotated into three types of genes, which accounted for 72.15% of total genes of *V. scophthalmi* strain VSc190401. Among them, 1327 genes were related to the cellular component, 1850 genes were related to the molecular function, and 1795 were related to the biological process. The GO terms with the highest numbers of genes in the classification of the biological process were oxidation-reduction process (174 genes, 5.46%) and regulation of transcription/DNA-templated (141 genes, 4.43%). The GO terms with the highest numbers of genes in the classification of cellular components were integral components of membrane (641 genes, 20.13%), cytoplasm (336 genes, 10.55%), and plasma membrane (91 genes, 2.86%). The GO terms with the highest numbers of genes in the classification of molecular function were ATP binding (281 genes, 8.82%), DNA binding (244, 7.66%), metal ion binding (127 genes, 3.99%), and transcription factor activity/sequence-specific DNA binding (82 genes, 2.57%). More information was shown in Fig. 5. Supplementary Table S2 lists all GO functional annotation information.

**Fig. 6:**
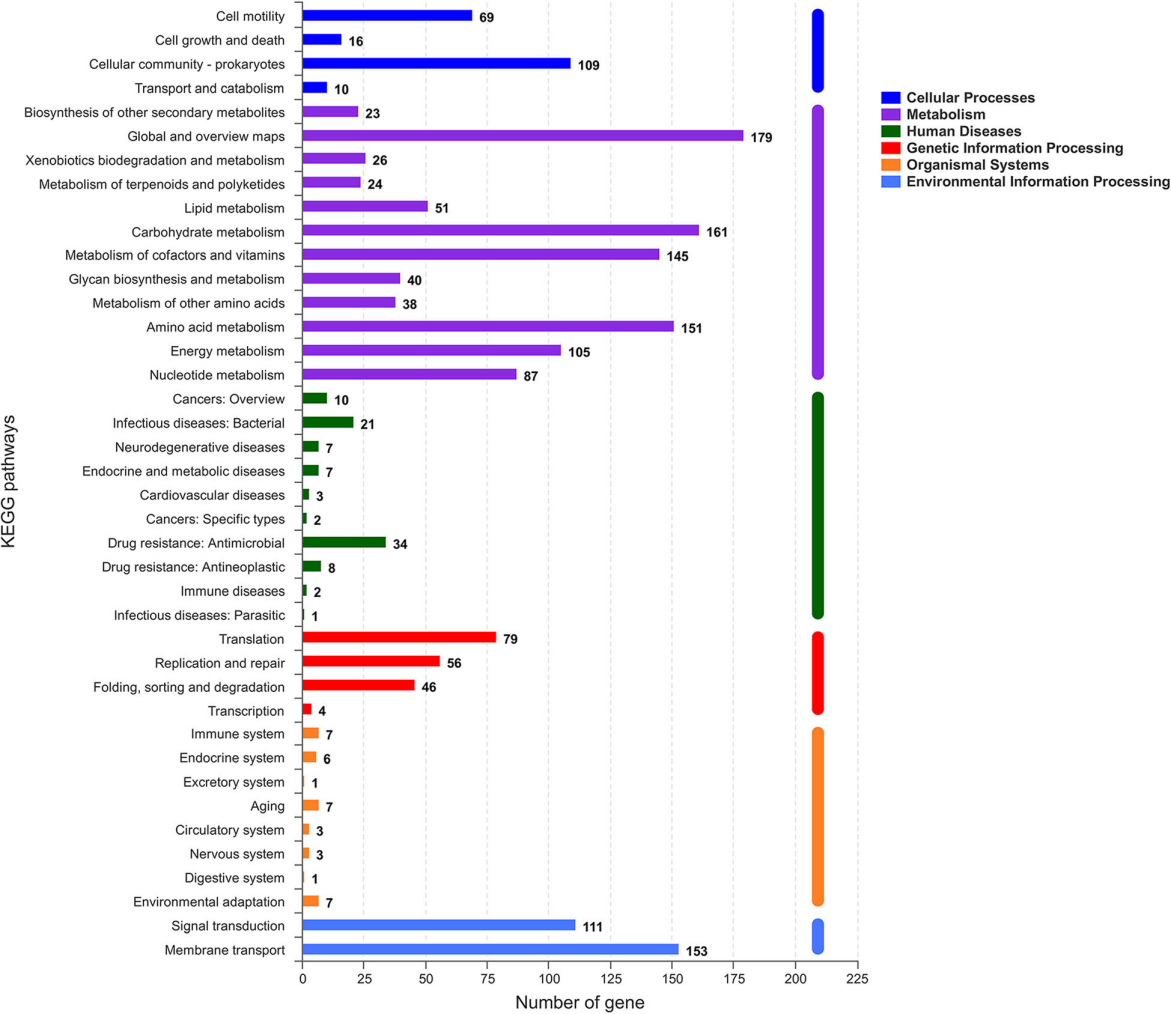
The KEGG pathway annotation results of *V. scophthalmi* strain VSc190401. The ordinate indicates the level 2 KEGG pathway classification, and the abscissa indicates the number of genes under the annotation of this classification. Different column colors represent the level 1 KEGG pathway classification. The rightmost bar indicates the number of genes under different level-1 classifications. Since the same gene may be annotated into multiple level-2 classifications, the number of genes classified by level 1 will be de-redundant

The results of the KEGG pathway analysis showed that 1915 genes were annotated into 196 known metabolic pathways. The metabolic pathway with the largest number of genes was the biosynthesis of amino acids, containing 106 genes, followed by two-component system (96 genes), carbon metabolism (83 genes), ABC transporters (81 genes), and purine metabolism (65 genes). Cluster analysis showed that 196 metabolic pathways were categorized into six classifications of cellular processes, metabolism, human diseases, genetic information processing, organismal systems, and environmental information processing, and the numbers of genes in these six classifications were 235, 1348, 112, 219, 47 and 268, respectively (Fig. 6). The 235 genes in the classification of cellular processes could be divided into four categories, and most of them were clustered into cell motility (74 genes) and cellular community-prokaryotes (135 genes). The 1348 genes in the classification of metabolism were divided into 12 categories. The categories with the largest gene number included global and overview maps (242 genes), carbohydrate metabolism (256 genes), amino acid metabolism (202 genes), and metabolism of cofactors and vitamins (149). The 112 genes in the classification of human diseases were clustered into 10 categories, and the categories with the largest gene number were drug resistance: antimicrobial (37 genes), and infectious diseases: bacterial (27 genes) and neurodegenerative diseases (13 genes). The 219 genes in the classification of genetic information processing were clustered into four categories. The categories with the largest gene number were translation (79 genes), replication and repair (90 genes), and folding, sorting and degradation (46 genes). The 47 genes in the classification of organismal systems were divided into eight categories. Among them, the top three categories with the largest gene number were the immune system (10 genes), aging (11 genes), endocrine system (nine genes), and environmental adaptation (seven genes). The environmental information was divided into two categories, including signal transduction (115 genes) and membrane transport (153 genes). Supplementary Table S3 shows all KEGG pathway annotation information.

Through the IslandViewer online system, 10 genomic islands were predicted to be contained in the whole genome of the strain Vsc190401. They were all located on chromosome I. The longest genomic island was 34,105 bp, and the shortest one was 7883 bp. Supplementary Table S4 and Supplementary Figure S1 illustrate their detailed information.

### Prediction of virulence genes of the strain VSc190401

To date, there are few studies on virulence factors of *V. scophthalmi*, and no specific virulence genes have been reported. A total of 334 potential virulence genes were predicted in the whole genome of the strain VSc190401. Moreover, 175 genes had annotation information in VFDB databases, including 107 offensive virulence genes, 31 defensive virulence genes, seven virulence-associated regulation genes, and 30 non-specific virulence genes (Table 1). Supplementary Table S5 shows detailed information.

**Table 1:** The annotation of virulence factors of the strain VSc190401 in VFDB databases

Virulence primary categories	Virulence Secondary categories	Gene numbers
Offensive virulence factors	Toxin	2
Offensive virulence factors	Secretion system	13
Offensive virulence factors	Adherence	81
Offensive virulence factors	Invasion	11
Defensive virulence factors	Cellular metabolism	1
Defensive virulence factors	Antiphagocytosis	21
Defensive virulence factors	Stress protein	9
Regulation of virulence-associated genes	Regulation	7
Nonspecific virulence factor	Iron uptake system	30

Among the offensive virulence genes, there were 81 genes related to adhesion, including flagella and pilus formation or motility exercise-related genes, such as *Flg*, *Fle*, *Flh*, *Fli*, *Tcp*, *PilB/D/G/H/R/T/U*, accessory colonization factor *AcfB/D* [18], adhesion proteins, *Lap*, *OmpU* [19] and so on. The results of comparative analysis to the NR database showed that the coverage of coding protein sequences was 96.44–100%, and the identity was 65.8–100%. There were 11 genes related to invasion, including flagella and T4SS genes. The coverage of their coding proteins was 56.14–100%, and the identity was 50–100% in the NR database. Moreover, 13 genes were related to secretion systems. Their sequence information was all similar to T3SS, T4SS, and T6SS of Gram-negative bacteria. The coverage of their coding proteins was 91.84–100%, and the identity was 56.7–100%. Two toxin genes were similar to *Cya* gene, the coding proteins of which were suspected as calmodulin-sensitive adenylate cyclase-haemolysin bifunctional protein [20]. The identity of their coding proteins was 98–99.7%.

The defensive virulence genes included 21 antiphagocytosis-related genes, nine stress protein-related genes, and one cellular metabolism-related gene. The antiphagocytosis-related genes included two categories of capsule genes (*Cpa*, *Cps*) [21, 22] and alginate genes (*algB/Q/U/R/Z*, *MucA*) [23, 24]. The identity of their coding proteins was 85.6–100%. The stress protein-related genes included superoxide dismutase enzyme gene (*sodB*), ATP-binding cassette transporter gene (*MntABC*), DNA repair protein gene (*RecN*), and respiratory metabolism gene (*ClpCP*) [25]. The identity of the protein sequence was 99–100%. The cellular metabolism-related genes were isocitratelyase coding gene fragments, with a coverage of 100% and an identity of 100%.

Among the non-specific virulence genes, the most were ATP-binding cassette transporter genes (20 genes, 98.4–100% protein sequence identity) and bacitracin-related genes (six genes, 90–100% protein sequence identity). Moreover, iron uptake system-related genes, such as *FbpABC* and *FeoAB* (99.4–100% protein sequence identity), were also found [26, 27].

The virulence-associated regulation genes contained two types of genes, *RelA* and *PhoP*. NR database comparative analysis results showed that the coverage of coding protein sequences was 100%, and their identity was 88.4–100%. Other studies have confirmed that *RelA* and *Phop* gene regulate the synthesis of bacterial virulence factors as well as their primary and secondary metabolites, thus affecting the bacterial pathogenicity [28, 29]. In addition, the virulence-related gene sequences of *ompA* (identity 90–100%), hemolysin (identity 96.7–100%), beta-hemolysin/cytolysin (identity 98.2–100%), enterobactin (identity 99.8–100%), and T2SS (identity 97.2–100%) were also found in the whole genome of the strain VSc190401.

Further analysis showed that the strain VSc190401 contained 36 secretion system-related genes, including one type I, 11 type II, six type IV, four type VI, 11 Sec-SRP pathway and three twin-arginine targeting (Tat) pathway. The analysis results also indicated that the strain VSc190401 only had a complete type II secretion system (the detailed information was shown in Supplementary Figure S2). This finding suggested that the type II secretion system was probably the only one product export pathway.

### The drug resistance phenotype and genotype analysis of the strain VSc190401

A total of 38 antibiotics belonging to 10 categories were selected to test the antimicrobial phenotype of strain VSc190401 through the Kirby-Bauer disk diffusion method. The antibiotics of 10 categories were β-lactam, aminoglycosides, macrolides, tetracyclines, polypeptides, quinolones, sulfonamides, nitrofurans, amphenicols, and others. The results showed that the strain VSc190401 was resistant to all aminoglycosides (including neomycin, streptomycin, kanamycin, gentamicin, amikacin), macrolides (including erythromycin, azithromycin, clarithromycin, acetylspiramycin) and amphenicols (including chloramphenicol and florfenicol). It was sensitive to polypeptides (polymyxin B), quinolones (including pipemidic, nalidixic, fleroxacin, lomefloxacin, ciprofloxacin, ofloxacin, norfloxacin, enrofloxacin), sulfonamides (sulfamethoxazole) and nitrofurans (including furazolidone). In β-lactam antibiotics, the strain showed resistance to cefoperazone, ceftizoxime, cefotaxime, ceftriaxone, ceftazidime, cefradine and oxacillin, while it was sensitive to penicillin, ampicillin, cefalexin and cefazolin. In tetracyclines antibiotics, the strain showed resistance to doxycycline, while it was sensitive to minocycline and tetracycline. Among other antibiotics, the strain showed resistance to rifampicin, while it was sensitive to novobiocin.

The results of Blast analysis in the CARD database showed that 180 drug resistance genes belonging to 27 categories were found in the whole genome of the strain VSc190401 (Table 2). In terms of drug resistance phenotype and genotype correlation, the strain VSc190401 contained the streptomycin-resistant genes, *gidB* and *vatB* [30], and rifampin-resistant genes, *rpoB* [31]. The phenotype was consistent with the genotype. The novobiocin-resistant genes, *alaS* and *cysB* [32], nalidixic-resistant genes, *gyrA/B* and *parC/E* [33], tetracycline-resistant genes, *tet31/34/35/B/R/S/T* and *adeR* [34], ciprofloxacin-resistant genes, *patA/B* [35], and bpolymyxin B-resistant genes, *PmrA/C/E*, *LpxA/C* and *rosB* [36], were found in the whole genome, while the strain was sensitive to these drugs. The phenotype was inconsistent with the genotype. Besides, the multiple resistance genes and complex genes of different antibiotics were also found in the strain, such as multiple resistance genes, *drrA* [37], *cpxA* and *ompR* [38], multidrug efflux pump systems, including ABC transporter superfamily of proteins *msbA* [39]; two-component signal transduction system, *EvgAS* [40]; two-component regulatory system, *BaeSR* [41]; an activator of *mtrCDE* multidrug-resistance efflux pump, *mtrA* [42]; MexEF-Opr multidrug efflux systems [43]; two-component regulatory systems, *VanRS* [44] and *ArlRS* [45]. These results showed that the resistance mechanism of the strain VSc190401 might be complex. Phenotypes of multiple drug resistance without specific resistance genes suggested that there were multiple drug metabolism pathways. Supplementary Table S6 shows the annotation information of all drug resistance genes.

**Table 2:** The drug-resistance genes annotation of the strain VSc190401 in CARD databases

Drug resistance categories	Gene numbers	Drug resistance categories	Gene numbers
Pleuromutilin antibiotic	3	penem	3
Carbapenem	7	phenicol antibiotic	12
Sulfonamide antibiotic	5	rifamycin antibiotic	4
Aminoglycoside antibiotic	10	isoniazid	3
Macrolide antibiotic	45	triclosan	11
Glycopeptide antibiotic	4	acridine dye	10
Tetracycline antibiotic	36	peptide antibiotic	14
Monobactam	5	lincosamide antibiotic	1
Diaminopyrimidine antibiotic	4	fluoroquinolone antibiotic	38
Streptogramin antibiotic	5	nitroimidazole antibiotic	6
Glycylcycline	5	penam	27
Sulfone antibiotic	3	cephalosporin	10
Aminocoumarin antibiotic	9	nybomycin	1
Cephamycin	9		

### Predictive analysis of pathogen-host interaction between the strain VSc190401 and host

According to the annotation results of the PHI database, the strain VSc190401 contained 518 genes related to pathogen-host interaction. Among them, there were 346 genes related to reduced virulence, 44 genes related to loss of pathogenicity, 36 genes related to hypervirulence, 12 genes related to lethal factors, 11 genes related to effector, three genes related to chemical resistance, and two genes related to chemical sensitivity, and there were 108 genes with unaffected pathogenicity. The hypervirulence and effector genes were the key genes in correlation with pathogenicity. The hypervirulence of the strain VSc190401 included T3SS function genes, *esaN* and *GdpX1*, virulence regulatory factors, *MorA*, *ccpE*, *RsmA*, *raxP* and *CdpR* [46–49], two-component sensor kinase, *BfiS* [50], histidine kinase and response regulator two-component system, *TcrX/Y* [51], and iron ion transporters, *feoB* and *pchD*. The effector genes of the strain VSc190401 included T6SS components, *VgrG* and *clpV* [52], phospholipase D effector, *LpdA* [53], pleiotropic effector of virulence synthesis and pathogenicity attenuation, *RpiRc* [54]. Supplementary Table S7 lists the detailed annotation information of pathogen-host interaction genes.

## Conclusions

The whole genome of pathogenic *V. scophthalmi* strain VSc190401 was 3,541,838 bp in length, including two circular chromosomes with the sizes of 3,286,294 bp and 202,664 bp, and two plasmids with the sizes of 24,538 bp and 28,342 bp, and this genome contained 3185 coding genes. The result of gene functional annotation indicated that 2648 genes were annotated into 22 types of genes in the COG database, accounting for 83.14% of total genes. Moreover, 2298 genes were annotated into three classifications in the Blast2GO database, accounting for 72.15% of total genes. Besides, 1915 genes were annotated into 196 known KEGG metabolic pathways, accounting for 60.13% of total genes. The analysis results based on the VFDB database showed that the strain VSc190401 contained 334 potential virulence genes, including four secretion systems of T1SS, T2SS, T4SS, T6SS related genes, and many different reported virulence genes. However, it only had one complete T2SS secretion system. Blast results in the CARD database showed that the strain contained 180 drug resistance genes belonging to 27 antibiotic resistance categories. However, many phenotypic resistance antibiotics were not related to drug resistance genes in the whole genome. Comparison analysis with the PHI database obtained 809 genes related to pathogen-host interaction, including a variety of regulatory factors or regulatory systems, as well as T3SS and T6SS functional genes. The whole-genome analysis suggested that pathogenic *V. scophthalmi* strain VSc190401 might have a complex molecular mechanism of drug resistance and pathogenicity, which need to be further explored in-depth research.

## Materials and methods

### The strain

*V. scophthalmi* strain VSc190401 was isolated from the liver of diseased half-smooth tongue sole (*Cynoglossus semilaevis*) cultured in an indoor farm. The symptoms of the diseased fish were hydrops in the abdomen and intestine, as well as enteritis. They were anesthetized and dissected by aseptic manipulation. Subsequently, their liver and intestine were sliced and homogenized with sterilized NaCl solution (1.5%), which were then cultured on a tryptic soy broth (TSB) agar medium plate containing 1.5% NaCl using streaking inoculation method. All plates were incubated at 28 °C for 24 h to 36 h to observe colony morphology. The largest single colonies with identical forms were considered as dominant bacteria. One of them was picked out for purification culture and used in further experiments.

The isolated strain was purified on TSB medium three times. The purified strain was used to prepare the living bacterial suspension at a density of 1.0 × 10^6^ CFU/mL. The strain pathogenicity was determined through an artificial challenge experiment by intraperitoneal injection. Half-smooth tongue sole and turbot with an average body weight of 50 g were purchased from a farm as the experimental animals. A total of 90 half-smooth tongue sole and 90 turbot were cultured in an indoor aquatic experiment system for 10 days to check their health firstly. Subsequently, the healthy fish were divided into six groups with 30 fish in each group, including one experimental group, one negative control group, and one blank control group of half-smooth tongue sole, and one experimental group, one negative control group, and one blank control group of turbot. Each group of fish was cultured in a 300-l aerated seawater tank. The experimental groups were intraperitoneally injected with bacterial suspension, the negative control group was injected with 1.5% sterile NaCl solution, and no injection was given in the blank control group. The injection dose was 0.1 mL bacterial suspension per 50 g body weight. During the experiment, the water was changed by 30% every day, and the temperature was maintained at 19 ± 1 °C. Fish were fed once a day.

When the fish in the experimental group developed symptoms, the dominant bacteria were isolated from the lesions, followed by purification and identification using the TSB agar medium plate [55]. After the isolated strain was identified as *V. scophthalmi* strain, its whole genome was sequenced on the Illumina HiSeq X platform. Meanwhile, the antibiotic resistance phenotype of this strain was detected using the Kirby-Bauer disk diffusion method. A total of 38 antibiotics were tested.

After the artificial challenge experiment finish, all the experimental fish in the negative control groups and blank control groups were returned to farm for continue farming. The fish in experimental groups were hypothermal shocked with ice-water mixture, until anesthesia. Subsequently, carbon dioxide was aerated in to the water to suffocate these fish to death. The dead fish were soaked in chlorine dioxide disinfectant at concentration of 1 × 10^− 4^ mg/L for 3 hours, then they were sent to the experimental waste disposal station for harmless treatment.

### Genomic DNA extraction, sequencing, and assembly

Genomic DNA of *V. scophthalmi* strain VSc190401 was extracted using Wizard® genomic DNA purification kit (Promega Biotechnology Co., Ltd., Beijing, China). The quality of extracted DNA was tested using a Qubit fluorometer (Thermo Fisher Scientific, Waltham, MA, USA) and a Nanodrop spectrophotometer (Thermo Fisher Scientific, Waltham, MA, USA). The purified genomic DNA was quantitatively analyzed by the TBS-380 fluorometer (Turner BioSystems Inc., Sunnyvale, CA, USA). Qualified genomic DNA was cut into > 10-kb fragments via G-tubes (Covaris Inc., Woburn, MA, USA) and used to construct the SMARTbell DNA database after terminal repair. Genome sequencing was conducted by Majorbio Biotechnology Co., Ltd. (Shanghai, China) using the Illumina HiSeq X sequencer. The obtained SMRT original sequence data were de novo assembled by hierarchical genomic assembly process (HGAP), and the quality of the newly assembled genome was corrected and verified by overlap layout consensus (OLC) and Quiver consensus algorithm to obtain the whole genome [56, 57].

### Genome component analysis and functional annotation

Glimmer software was used to predict the coding sequences (CDSs) in the genome. RepeatMasker software was used to predict tandem repeat sequences [58]. The rRNAs and tRNAs belonging to non-coding RNAs were predicted by rRNAmmer software [59] and tRNAscan software [60], respectively. Furthermore, different databases, such as Gene Ontology (GO), Kyoto Encyclopedia of Genes and Genomes (KEGG), Cluster of Orthologous Groups (COG), Non-Redundant Protein Sequence Database in NCBI (NR), UniProt/Swiss-Prot, Pfam, Virulence Factors of Pathogenic Bacteria (VFDB), Pathogen Host Interactions (PHI) and the Comprehensive Antibiotic Research Database (CARD), were used to annotate the functions of coding genes. Finally, the genome circle map was drawn and annotated using the Circos software (http://circos.ca/). The genomic islands were predicted through IslandViewer online system (http://www.pathogenomics.sfu.ca/islandviewer/upload/).

### Phylogenetic analysis

The complete reference genome sequences were downloaded from the NCBI database, and comparative analysis was performed based on the average nucleotide identity (ANI) value described by Richter and Rossello [61]. The OAT software (https://www.ezbiocloud.net/tools/orthoani) was used for ANI analysis [62]. The bacterial strains of the reference genome were as follows: *V. scophthalmi* VS-12, *V. scophthalmi* VS-05, *V. cholerae* RFB05, *V. anguillarum* ATCC-68554, *V. rotiferianus* CAIM 577, *V. harveyi* ATCC 33843, *V. parahaemolyticus* FORC_008 and *V. vulnificus* ATCC 27562.

## Supplementary Information

**Additional file 1:** **Figure S1. **Genome islands linear map.

**Additional file 2:** **Figure S2.** The secretory system pathway.

**Additional file 3:** **Table S1.** The COG functional annotation information.

**Additional file 4:** **Table S2.** The GO functional annotation information.

**Additional file 5:** **Table S3.** The KEGG pathway annotation information.

**Additional file 6:** **Table S4. **The gene information of genomic islands on chromosome I.

**Additional file 7:** **Table S5.** The virulence factors annotation information.

**Additional file 8: Table S6.** The drug-resistance genes annotation information.

**Additional file 9:** **Table S7.** The PHI genes annotation information.

## Data Availability

The whole genome sequence of *Vibrio scophthalmi* strain VSc190401 has been deposited in the NCBI GenBank server under the SRA accession number PRJNA628013 for chromosome 1, chromosome 2, and the plasmids.

## Abbreviations

COG: Clusters of Orthologous Groups of proteins
GO: Gene Ontology
KEGG: Kyoto Encyclopedia of Genes and Genomes
VFDB: Virulence Factors of Pathogenic Bacteria
NR: Non-Redundant Protein Sequence Database in NCBI
PHI: Pathogen Host Interactions
CARD: Comprehensive Antibiotic Research Database
HGAP: Hierarchical genomic assembly process
OLC: Overlap layout consensus
CODs: Coding sequences
ANI: Average nucleotide identity
