# Correction to: Complete genome analysis of a virulent Vibrio scophthalmi strain VSc190401 isolated from diseased marine fish half-smooth tongue sole, *Cynoglossus semilaevis*

**DOI:** 10.1186/s12866-020-02067-0

**Published:** 2020-12-14

**Authors:** Zheng Zhang, Yong-xiang Yu, Yin-geng Wang, Xiao Liu, Li-fang Wang, Hao Zhang, Mei-jie Liao, Bin Li

**Affiliations:** 1 Key Laboratory of Maricultural Organism Disease Control, Ministry of Agriculture and Rural Affairs, Yellow Sea Fisheries Research Institute, Chinese, Academic of Fishery Sciences, Qingdao, Shandong 266071 PR China; 2 Laboratory for Marine Fisheries Science and Food Production Processes, Pilot National Laboratory for Marine Science and Technology (Qingdao), Qingdao, Shandong 266237 PR China

**Correction to: BMC Microbiol. 20, 341 (2020)**

**https://doi.org/10.1186/s12866-020-02028-7**

Following publication of the original article [1], we were notified that Prof. Yin-geng Wang needs to be marked as the second corresponding author.

The original article has been corrected.
