# Supplementary figures and images for: Complete genome analysis of a virulent Vibrio scophthalmi strain VSc190401 isolated from diseased marine fish half-smooth tongue sole, Cynoglossus semilaevis
Source: BMC Microbiol. 2020 Nov 11;20:341. doi: 10.1186/s12866-020-02028-7 (PMC7661262; doi:10.1186/s12866-020-02028-7)

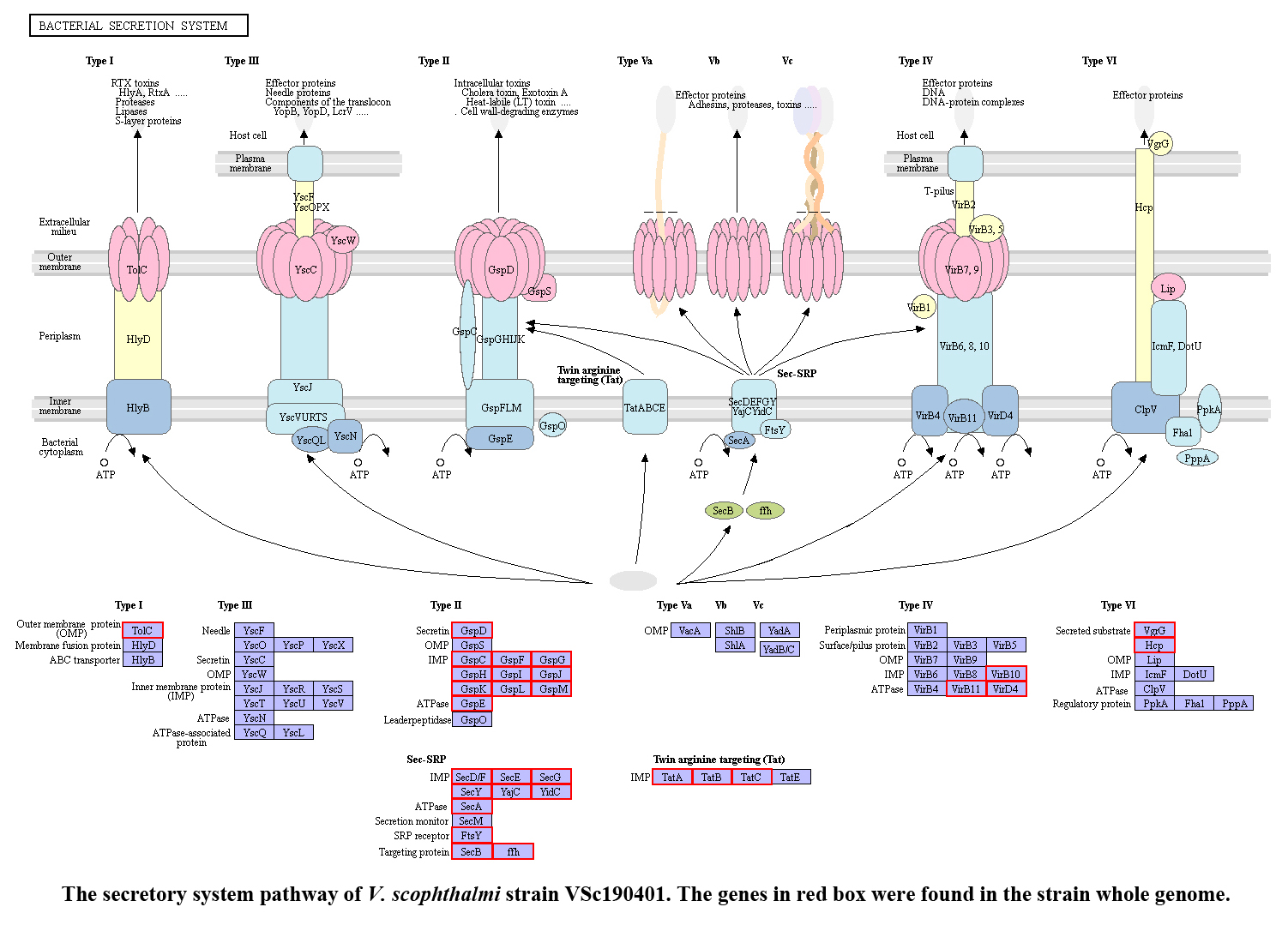

Supplement: Supplementary file 2 — Additional file 2: Figure S2. The secretory system pathway. [file 12866_2020_2028_MOESM2_ESM.jpg]
